# Variations in hepatic circulation: a study of 500 abdominal computed tomography scans

**DOI:** 10.1590/1677-5449.202401102

**Published:** 2025-05-30

**Authors:** Maria Eduarda Zen Biz, Jéssica Paola Salame, Gustavo Gumz Correia, Rafael Saviolo Moreira

**Affiliations:** 1 Centro Universitário de Brusque – UNIFEBE, Brusque, SC, Brasil.; 2 Universidade Federal do Paraná – UFPR, Curitiba, PR, Brasil.; 3 Universidade Federal de São Paulo – UNIFESP, São Paulo, SP, Brasil.

**Keywords:** anatomic variation, liver circulation, portal vein, hepatic artery, tomography

## Abstract

**Background:**

Knowledge of the vascular anatomy of the liver and other abdominal organs helps surgeons improve preoperative planning, achieve greater surgical success, prevent complications, and reduce morbidity and mortality.

**Objectives:**

To report the prevalence of anatomical variation in the proper hepatic artery and portal vein observed through computed tomography.

**Methods:**

This retrospective study was based on 500 3-phase abdominal computed tomography scans. Variations in arterial anatomy were classified according to the Michels system (1966), while those in regarding portal vein anatomy were classified according to the Cheng system (1996).

**Results:**

A total of 31.2% of the cases showed variations in arterial vascularization, the most prevalent being type V (8.2%). No participants were identified with type X, and 0.4% could not be classified. A total of 21.8% showed variation in venous vascularization, with type IV being the most prevalent (8%).

**Conclusions:**

Medical knowledge of these variations and their prevalence is fundamental for the correct surgical management of upper abdomen pathologies and lower rates of postoperative complications. Variations not classified by previous trials should be categorized according to their clinical importance, and new studies should clarify national population patterns to reduce mortality rates from surgical procedures that involve these vessels.

## INTRODUCTION

Knowledge of the vascular anatomy of the upper abdomen, both normal and abnormal, is essential for quality health care, especially invasive surgical or radiological procedures in the hepatic hilum.^1-4^ Abnormalities in the arteries and veins are frequently found in imaging tests or cadaver dissections. They are generally considered to be of embryological origin and they can manifest as absent, duplicate, accessory, replaced, etc.^5,6^ Variations of the proper hepatic artery (HA) and portal vein (PV), which are essential vessels for the adequate blood supply to the liver, have been studied for decades by anatomists, since they affect between 0.1% and 47.8% of the population.^4,7-10^

In classical anatomy, the celiac trunk branches off from the abdominal part of the aorta and then divides into the left gastric, splenic, and common HAs. The latter divides into the gastroduodenal, right gastric, and proper HAs. The proper HA, located anterior to the PV and contained in the hepatoduodenal ligament, irrigates approximately 25% of the liver through the left and right hepatic branches.^1-3,11,12^

The anatomical variations of the hepatic arterial system can be explained embryologically by the persistence of segments that should be discontinued, by the regression of vessels that should persist, or by both simultaneously.^13^ Among all possible variations of the hepatic pedicle, arterial variations are the most frequent, representing between 13% and 45% of the cases reported in the literature. ^2,4,7-10^

**Figure 1:**
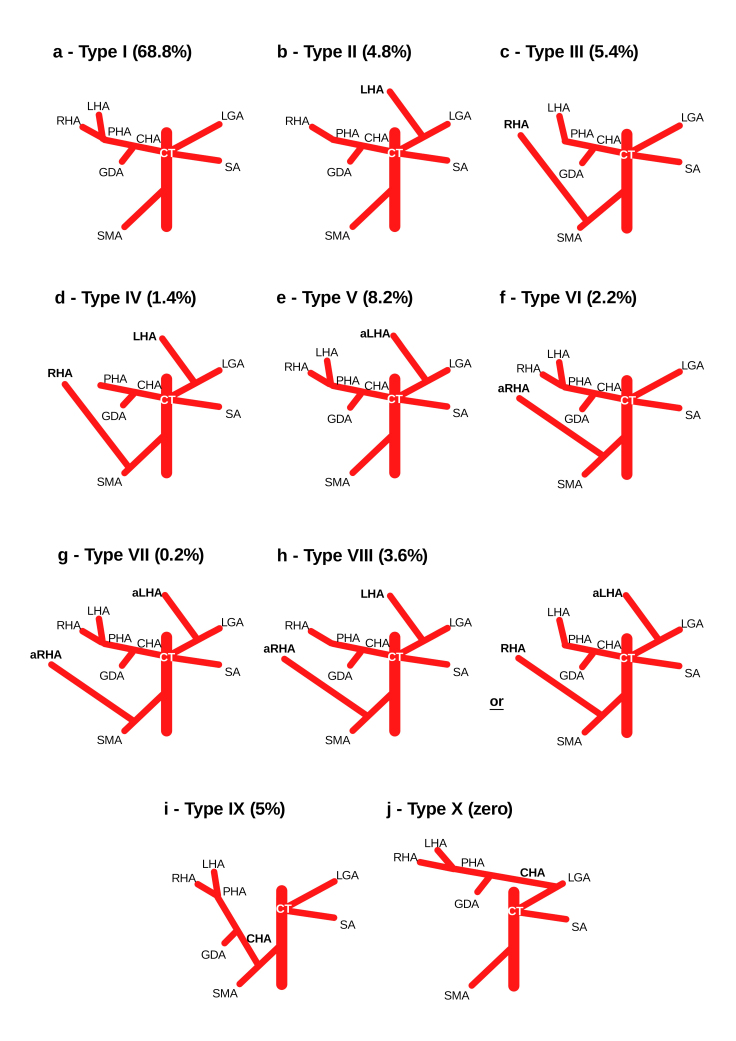
Schematic illustration of variations in hepatic vascularization observed in this study. PHA = proper hepatic artery; CHA = common HA; LHA = left HA; aLHA = accessory LHA; RHA = right HA; aRHA = accessory RHA; LGA = left gastric artery; SMA = superior mesenteric artery; CT = celiac trunk; SA = splenic artery; GDA = gastroduodenal artery.

In 1966, after 200 dissections in cadavers, Michels^14^ established an internationally recognized classification system involving 10 HA variations: type I - normal anatomy; type II - left HA (LHA) originating from the left gastric artery (LGA); type III - right HA (RHA) originating from the superior mesenteric artery (SMA); type IV - the coexistence of types II and III; type V - accessory LHA originating from the LGA; type VI - accessory LHA originating from the SMA; type VII - coexistence of types V and VI; type VIII - replaced LHA originating from the LGA plus accessory LHA originating from the SMA or accessory LHA originating from the LGA plus replaced LHA originating from the SMA; type IX - common LHA originating from the SMA; and type X - common HA originating from the LGA (Figure 1).^7,9,13,15^

The hepatic portal system receives blood from a large part of the gastrointestinal tract, as well as from the accessory glands (pancreas and gallbladder) and the spleen, directing it to the hepatic sinusoids.^12^ The PV, responsible for approximately 75% of hepatic vascularization, originates at the junction of the splenic and superior mesenteric veins, forming a main trunk that bifurcates into right and left branches.^13,16,17^

In terms of prevalence, anatomical variations in the venous component of the hepatic hilum are the most frequent, ranging from 0.1% to 47.8% of previously described cases. ^2,9,12,17-19^

**Figure 2:**
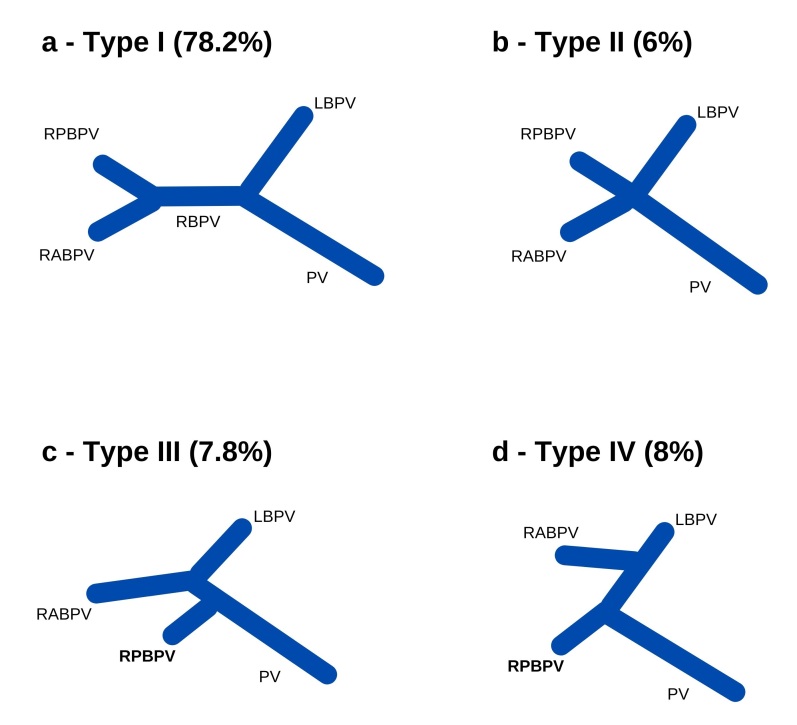
Schematic illustration of variations in the intrahepatic portal system observed in this study. PV = main trunk of the portal vein; LBPV = left branch of the PV; RBPV= right branch of the PV; RABPV = right anterior branch originating directly from the PV; RPBPV = right posterior branch originating directly from the PV.

The Cheng classification system (1996) separates PV types into: type I - bifurcation into left and right branches, with the latter dividing into anterior and posterior branches; type II - trifurcation of the PV; type III - early origin of the right posterior branch, with subsequent bifurcation of the main trunk into right and left anterior branches; type IV - right anterior branch originating from the left branch of the PV (Figure 2).^7,13,18,20^

In the present study, we used the 1966 Michels classification system, since it specifically establishes the difference between replaced and accessory arteries, important concepts from a surgical point of view.^1,21^ In 1996, Cheng divided variations of the intrahepatic branches of the PV into 4 classes.^1^

Regarding anatomical variations, replaced arteries are so named because the normal artery is absent and they can supply an entire hepatic lobe on their own. However, accessory or aberrant arteries are additional, non-essential arteries that are functionally responsible for only part of a lobe.^5^

Regarding their clinical implications, complications, such as segmental or lobar hepatic necrosis, which result from the inadvertent ligation of one of these vessels (especially replaced arteries), can be avoided by recognizing such anatomical variations. Such discernment is even more relevant in videolaparoscopy, cadaveric or live liver transplants, and in the recent increase in gastroplasties.^3,5,6,22^

Misunderstanding changes in the intrahepatic course of the PV (eg, PV embolization, hepatectomies, organ donation for transplantation, etc.) can impair the surgical technique or even make surgeries and procedures unfeasible.^7,12,18,23^

To assess liver anatomy, technological advances in morphological or imaging studies have allowed more research participants, since these methods are faster than cadaver dissection techniques.^6^ Furthermore, such examinations enable better planning for liver surgeries and, thus, lower morbidity and mortality rates.^4,22^

The gold standard for assessing vascular structures is angiography. However, computed tomography (CT) has proven to be a faster and less expensive alternative that can obtain relevant clinical data. Furthermore, different techniques, such as maximum intensity projection, multiplanar reconstruction and 3-dimensional reconstruction, can be applied to facilitate the characterization of vessels and, consequently, anatomical variations.^9,12,19^

Due to the complexity of hepatic irrigation, the great arterial and venous variability, the importance of access to these vessels, and the scarcity of studies correlating the structures evaluated in 3-phase CT, our objective was to evaluate the presence and the association of arterial and portal hepatic variations in the local population.

## METHODOLOGY

This basic retrospective study with a descriptive quantitative approach examined the medical records of 500 Brazilian adults who underwent a 3-phase abdominal CT scan after adequate medical evaluation (for reasons unrelated to the study) at a hospital in the midwestern region of the state of Santa Catarina, Brazil between January 2021 and June 2022.

This study was approved by the institutional research ethics committee (decision 5,616,116; certificate 61836122.7.0000.5636), which waived the requirement for informed consent. The inclusion criteria were individuals of either sex aged ≥ 18 years who underwent abdominal CT for various clinical indications. The exclusion criteria were individuals whose extreme abdominal abnormalities made it difficult to assess the hepatic vascular network.

All images were obtained from the authors’ personal archives using a 16-channel multidetector CT scanner (Somaton Emotion, Siemens AG, Munich, Germany). The examination protocol consisted of 1-mm axial slices with a pitch of 1.0. The contrast agent was Omnipaque 350 (active ingredient iohexol; GE Healthcare, Chicago, IL, USA), used at a concentration of 755 mg/ml and administered intravenously using an injection pump (MEDRAD Vistron, Bayer AG, Leverkusen, Germany) at a flow rate of 3.5 ml/s with bolus tracking delay time. The standard field of view was 250 mm. The reconstruction thickness of the images was 1 mm. The images were exported in standard DICOM format and processed in OsiriX MD (Pixmeo SARL, Bernex Switzerland) and RadiAnt (Medixant, Poznan, Poland).

The anatomy of the celiac trunk, LGA, splenic artery, common HA, proper HA, RHA, LHA, SMA, accessory hepatic arteries, PV, and the left and right branch of the PV were analyzed. The images were classified by a radiologist with 10 years of experience. To determine the arterial pattern, analyses were performed in the axial plane, multiplanar reconstruction techniques were performed in the coronal and sagittal planes, as well as 3-dimensional reconstructions in maximum intensity projection.

Anatomical normality for the arterial system was considered a proper HA originating from the common HA and bifurcating into the right and left hepatic arteries, without accessory arteries for arterial vascularization. Anatomical normality for the venous system was considered bifurcation of the main trunk of the PV into left and right branches, with the latter dividing into the anterior right and posterior right branches for portal vascularization. Arterial and portal vein variations were classified according to the Michels^14^ and Cheng et al.^24^ systems, respectively. All observed data were manually organized into tables in Google Sheets, which generated automatic graphs that were visually analyzed by the researchers.

## RESULTS

Regarding sample characterization, 307 (61.4%) were women and 193 (38.6%) were men. The mean age of the participants was 50.4 (SD, 16.7) years (minimum of 18, maximum 92). HA anatomy and variation were classified according to the Michels system (1966) (Table 1), while PV anatomy and variation were classified according to the Cheng et al.^24^ system (Table 2).

**Table 1:** Hepatic artery anatomy according to the Michels classification system^14^ .

**Type**	**Description**	**Female**		**Male**		**Total**	
		**n**	**%**	**n**	**%**	**n**	**%**
I	Normal	207	67.6	137	71.4	344	68.8
II	LHA from LGA	17	5.6	7	3.6	24	4.8
III	RHA from SMA	18	5.9	9	4.7	27	5.4
IV	II and III	3	1.0	4	2.1	7	1.4
V	Accessory LHA from LGA	22	7.2	19	9.9	41	8.2
VI	Accessory RHA from SMA	7	2.3	4	2.1	11	2.2
VII	V and VI	0	0.0	1	0.5	1	0.2
VIII	III and V or II and VI	11	3.6	7	3.6	18	3.6
IX	CHA from LGA	21	6.9	4	2.1	25	5.0
X	CHA from SMA	0	0.0	0	0.0	0	0.0

LHA = left hepatic artery; LGA = left gastric artery; RHA = right hepatic artery; SMA = superior mesenteric artery; CHA = common hepatic artery. Source: The authors.

**Table 2:** Portal vein anatomy according to the Cheng et al.^24^ classification system.

**Type**	**Description**	**Female**		**Male**		**Total**	
		**n**	**%**	**n**	**%**	**n**	**%**
I	Bifurcation into left and right branches with the latter dividing into anterior and posterior branches	237	77.5	153	79.7	390	78.2
II	PV trifurcation	17	5.6	13	6.8	30	6.0
III	Early bifurcation of the right anterior and posterior branches. with the left PV arising from the right posterior branch	25	8.2	13	6.8	38	7.8
IV	Right posterior branch originating from the left branch of the PV	27	8.8	13	6.8	40	8.0

PV = portal vein. Source: The authors.

Normal anatomical distribution of the HA, or Michels type I (Figure 1a), is a common HA which emits a proper HA that bifurcates into LHA and RHA. This was the predominant type in this study, corresponding to 344 (68.8%) cases. Type V (Figure 1e) was the second most predominant type, observed in 41 (8.2%) cases. Types III (Figure 1c), IX (Figure 1i), and II (Figure 1b) followed, with 27 (5.4%), 25 (5%), and 24 (4.8%) cases, respectively.

None of the samples were identified as type X (Figure 1j), and 2 (0.4%) participants could not be classified. One corresponded to a case of RHA originating directly from the aorta, and the other to a case simultaneously involving a common HA originating from the SMA and accessory LHA originating from the LGA.

Regarding venous variations, 391 (78.2%) of the tomographies indicated a normal intrahepatic PV (ie, Cheng type I) (Figure 2a). The prevalence of types IV (Figure 2d) and III (Figure 2c) was similar, with 40 (8%) and 39 (7.8%) cases, respectively. Finally, type II (Figure 2b) was the rarest, with 30 (6%) occurrences.

In the overall sample, the proportion of PV type I (78.3%) (Table 2) was significantly higher than HA type I (69.1%) (Table 1) according to the χ^2^ test (p = 0.0009). When analyzing the sample according to sex, we found a non-significant proportion of men (PV ​​type I, 79.7%, and HA type I, 71.4%; p = 0.058) and a significant proportion of women (PV ​​Type I, 77.5%, and HA type I, 67.6%; p = 0.006). Finally, 258 (51.6%) individuals had normal HA and PV anatomy, while 33 (6.6%) had simultaneous variation in both.

## DISCUSSION

**Figure 3:**
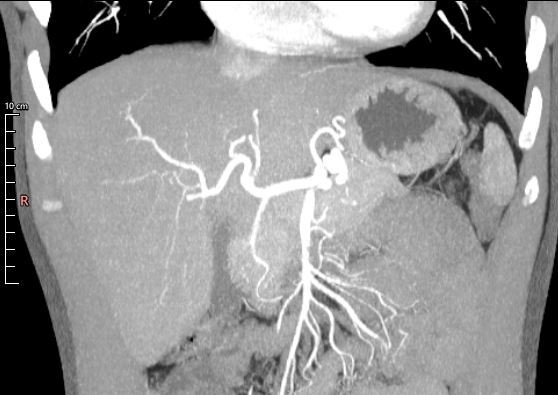
Abdominal computed tomography image of the abdomen reconstructed in maximum intensity projection in the arterial phase, coronal plane, showing a proper hepatic artery originating from a common hepatic artery and bifurcating into right and left hepatic arteries.

Other studies on the prevalence of HA variations have been published (Table 3). Dazzi et al.^25^ used angiotomography and intraoperative association to assess a sample of 66 liver donors, revealing results with 100% sensitivity and 100% specificity. Our Michels type I results (Figure 3) are comparable to theirs (68.8% and 68.2%, respectively).

**Table 3:** Comparative analysis of studies on the prevalence of hepatic artery variation.

**Michels type**		**This study (%)**	**Dazzi et al.** ^25^ **(%)**	**Fonseca-Neto et al.** ^8^ **(%)**	**Brasil et al.** ^4^ **(%)**	**Imam et al.** ^26^ **(%)**
I		68.8	68.2	86.8	82	60.9
II		4.8	10.6	2.7	1	9.1
III		5.4	10.6	5.6	10	7.4
IV		1.4	0	0.8	0	0.4
V		8.2	3	0.6	1	4.5
VI		2.2	0	0.4	1	0.4
VII		0.2	0	0.0	0	0.4
VIII		3.6	0	0.0	0	2.9
IX		5.0	3	0.0	4	0.8
X		0.0	0	0.0	0	0.0
Not classifiable		0.4	4.5	2.9	1	12.9

Source: The authors.

In 479 liver transplants, Fonseca-Neto et al.^8^ found a high prevalence of normal anatomy (86.8%) and a low prevalence of types II, III, and IV (2.7%, 5.6% and 0.8%, respectively), which was similar to our results (4.8%, 5.4% and 1.4%, respectively). Brasil et al.^4^ evaluated 100 angiotomography scans, finding prevalence rates of 1% for type VI and 4% for type IX. These results are also similar to ours (2.2% and 5%, respectively).

**Figure 4:**
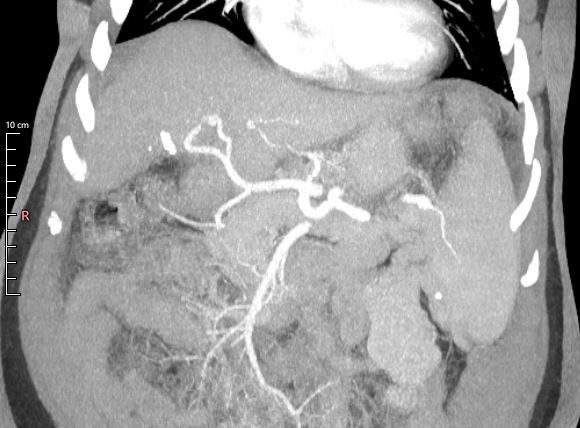
Abdominal computed tomography image with maximum intensity projection reconstructed in the arterial phase, coronal plane, showing an accessory left hepatic artery originating from the left gastric artery.

Finally, Imam et al.^26^ also found similar results to ours regarding HA types VII and VIII (0.4% and 2.9%, respectively vs 0.2% and 3.6%, respectively). However, their incidence of Michels type V was higher than ours (8.2% vs 4.5%) (Figure 4).

Of the 500 CT scans we analyzed, only 2 (0.4%) could not be classified according to the Michels system. In other studies, classification failure has been as high as 12.9% (Table 3).^26^ Since anatomical variations derive from embryological alterations, it is difficult to develop a standardized model that addresses all possibilities.^8^

Both of the unclassifiable conditions in the present study have been previously documented in the literature, ie, a RHA originating directly from the aorta and a common HA originating from the SMA with an accessory LHA simultaneously originating from the LGA. The first case is relevant due to the difficult access, requiring angiography and transarterial chemotherapy to treat hepatocarcinoma, while the second has been associated with increased complication rates after pancreaticoduodenectomy.^26^

We performed a comparative analysis of studies on intrahepatic PV variation (Table 4), finding that our sample had a higher incidence of structural variation (21.8%) than that of Lee et al.^27^ (14.5%), Anwar et al.^9^ (4.8%), or Vidya et al.^28^ (12.5%). Our sample also had a higher prevalence of Cheng type IV (8%) than these studies (1%, 0.8%, and 0%, respectively).

**Table 4:** Comparative analysis of studies on the prevalence of intrahepatic portal vein variation.

**Cheng type**	**This study (%)**	**Lee et al.** ^27^ **(%)**	**Anwar et al.** ^9^ **(%)**	**Vidya et al.** ^28^ **(%)**
I	78.2	85.5	95.2	87.5
II	6.0	7.1	1.6	10.0
III	7.8	6.4	2.4	2.5
IV	8.0	1.0	0.8	0.0

Source: The authors.

**Figure 5:**
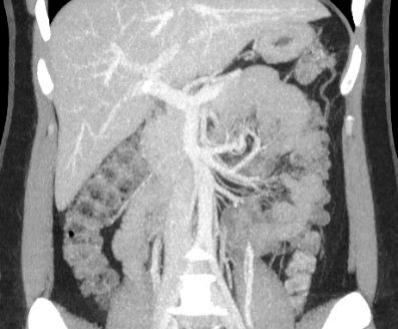
Abdominal computed tomography image in the portal phase, coronal plane, showing the right anterior branch of the portal vein originating from the left branch.

The clinical importance of the high prevalence of Cheng type IV (Figure 5) in our sample is due to the contraindication for certain surgical procedures, such as donation of the right hepatic lobe for organ transplantation or tumor resection, since it makes adequate reconstruction impossible.^9^ Type II was the most prevalent variation in Lee et al.^27^ and Vidya et al.^28^ (7.1% and 10%, respectively), while type III was the most prevalent (2.4%) in Anwar et al.^9^

## FINAL CONSIDERATIONS

This study found a high prevalence of variation in hepatic vascularization, both in the proper HA and the intrahepatic PV. Similar data have been found in other studies worldwide. Tomographic analysis and knowledge of the vascular anatomy of the liver and abdominal organs, both normal and abnormal, facilitate preoperative planning, lead to greater surgical success, prevent complications, and reduce morbidity and mortality. Variations not classified by previous trials should be categorized according to their clinical importance, and further Brazilian studies should be encouraged to clarify the patterns found in the national population.
