# Supplementary material for: Variations in hepatic circulation: a study of 500 abdominal computed tomography scans
Source: J Vasc Bras. 2025 May 30;24:e20240110. doi: 10.1590/1677-5449.202401102 (PMC12143226; doi:10.1590/1677-5449.202401102)
Supplement: Translation

# Translation: Variações anatômicas na circulação hepática: estudo em 500 tomografias computadorizadas de abdome

**DOI:** 10.1590/1677-5449.202401101

**Authors:** Maria Eduarda Zen Biz, Jéssica Paola Salame, Gustavo Gumz Correia, Rafael Saviolo Moreira

**Affiliations:** 1 Centro Universitário de Brusque – UNIFEBE, Brusque, SC, Brasil.; 2 Universidade Federal do Paraná – UFPR, Curitiba, PR, Brasil.; 3 Universidade Federal de São Paulo – UNIFESP, São Paulo, SP, Brasil.

**Keywords:** variação anatômica, circulação hepática, veia porta, artéria hepática, tomografia

## Abstract

**Contexto:**

O conhecimento sobre a anatomia vascular do fígado e outros órgãos abdominais auxilia o cirurgião a realizar um melhor planejamento pré-operatório, obter maior sucesso cirúrgico, prevenir complicações e diminuir a morbimortalidade.

**Objetivos:**

Relatar a prevalência de variações anatômicas da artéria hepática própria e da veia porta, observadas em exames de tomografia computadorizada.

**Métodos:**

Trata-se de um estudo retrospectivo no qual foram avaliados 500 exames de tomografia computadorizada de abdome trifásico. As variações foram classificadas de acordo com Michels (1966) para a anatomia arterial e Cheng (1996) para a portal.

**Resultados:**

Entre os casos estudados, 31,2% apresentaram variação quanto à vascularização arterial, sendo a mais prevalente a do Tipo V, com 8,2%. Nenhum participante foi identificado com o Tipo X, e 0,4% não puderam ser classificados. Com respeito à venosa, 21,8% eram variados, com maior prevalência do Tipo IV, observado em 8% das imagens analisadas.

**Conclusões:**

Pode-se agregar conhecimento médico sobre as variações descritas e demonstrar sua prevalência na população estudada, o que é fundamental para o correto manejo das patologias cirúrgicas do abdome superior e para a redução das taxas de complicações pós-operatórias. Variações não classificadas por ensaios prévios devem ser categorizadas conforme sua importância clínica. Além disso, novos estudos devem ser encorajados a fim de compreender melhor os padrões populacionais do país e reduzir as taxas de mortalidade em procedimentos cirúrgicos relacionados aos vasos citados.

## INTRODUÇÃO

O conhecimento da anatomia vascular do abdome superior, tanto em sua forma normal quanto em suas variações, é essencial para proporcionar uma assistência à saúde de qualidade, especialmente em procedimentos cirúrgicos ou radiológicos invasivos no hilo hepático^1-4^ . Anormalidades em artérias e veias são frequentemente encontradas durante exames de imagem ou em dissecções de cadáveres em laboratórios. Geralmente, estão associadas à sua origem embriológica e podem manifestar-se de forma ausente, duplicada, acessória ou substituta, entre outras^5,6^ . As variações de artéria hepática própria (AHP) e veia porta (VP), que são vasos indispensáveis para o adequado suprimento sanguíneo do fígado, são estudadas há décadas por anatomistas, uma vez que atingem entre 0,1 e 47,8% da população^4,7-10^ .

Na anatomia clássica, a parte abdominal da aorta emite o tronco celíaco, que se divide em artérias gástrica esquerda, esplênica e hepática comum. A última origina as artérias gastroduodenal, gástrica direita e hepática própria (HP). A HP, localizada anteriormente à VP e contida no ligamento hepatoduodenal, irriga cerca de 25% do fígado através dos ramos hepáticos esquerdo e direito^1-3,11,12^ .

As variações anatômicas do sistema arterial hepático podem ser explicadas embriologicamente pela persistência de segmentos que deveriam ser descontinuados, pela regressão de vasos que deveriam persistir ou por ambas as ocorrências simultaneamente^13^ . Entre todas as variações possíveis do pedículo hepático, as arteriais são as mais frequentes, representando entre 13 e 45% dos casos relatados na literatura^2,4,7-10^ .

Em 1966, Michels^14^ estabeleceu, após 200 dissecções em cadáveres, uma classificação reconhecida internacionalmente referente a 10 variações da artéria hepática (AH) normal, sendo: Tipo I - anatomia normal; Tipo II - artéria hepática esquerda (AHE) originando-se da artéria gástrica esquerda (AGE); Tipo III - artéria hepática direita (AHD) originando-se da artéria mesentérica superior (AMS); Tipo IV - coexistência dos tipos II e III; Tipo V - AHE acessória originando-se da AGE; Tipo VI - AHD acessória originando-se da AMS; Tipo VII - coexistência dos tipos V e VI; Tipo VIII - AHE substituta originando-se da AGE, AHD acessória originando-se da AMS ou AHE acessória originando-se da AGE, AHD substituta originando-se da AMS; Tipo IX - AH comum (AHC) originando-se da AMS; e Tipo X - AHC originando-se da AGE (Figura 1)^7,9,13,15^ .

**Figura 1:** Ilustração esquemática das variações de vascularização hepática encontradas neste estudo. AHP = artéria hepática própria; AHC = artéria hepática comum; AHE = artéria hepática esquerda; AHEa = artéria hepática esquerda acessória; AHD = artéria hepática direita; AHDa = artéria hepática direita acessória; AGE = artéria gástrica esquerda; AMS = artéria mesentérica superior; TC = tronco celíaco; AE = artéria esplênica; AGD = artéria gastroduodenal.

O sistema porta do fígado recebe sangue proveniente de vasta parte do trato gastrointestinal, bem como das glândulas acessórias (pâncreas e vesícula biliar) e baço, direcionando-o até os sinusoides hepáticos^12^ . A VP, responsável por aproximadamente 75% da vascularização hepática, tem origem na junção das veias esplênica e mesentérica superior, formando um tronco principal que se bifurca nos ramos direito e esquerdo^13,16,17^ .

Se tratando de prevalência, as variações anatômicas no componente venoso do hilo hepático são as mais frequentes, variando de 0,1 a 47,8% de casos descritos previamente^2,9,12,17-19^ .

A classificação de Cheng, de 1996, separa os tipos de VP em: Tipo I - bifurcação em ramos esquerdo e direito, com o último dividindo-se nos ramos anterior e posterior; Tipo II - trifurcação da VP; Tipo III - origem precoce do ramo posterior direito, com posterior bifurcação do tronco principal em ramos anterior direito e esquerdo; Tipo IV - ramo anterior direito originando-se do ramo esquerdo da VP (Figura 2)^7,13,18,20^ .

**Figura 2:** Ilustração esquemática acerca das variações do sistema porta intra-hepático encontradas neste estudo. VP = tronco principal da veia porta; VPE = veia porta ramo esquerdo; VPD = veia porta ramo direito; VPDA = veia porta ramo direito anterior; VPDP = veia porta ramo direito posterior.

No presente estudo, optou-se por utilizar a classificação de Michels, de 1966, uma vez que esta estabelece de forma específica a diferença entre artérias substitutas e acessórias, conceitos importantes do ponto de vista cirúrgico^1,21^ . Já as variações dos ramos venosos intra-hepáticos portais foram estudadas em 1996 e divididas em quatro classes por Cheng^1^ .

Em relação às variações anatômicas, as artérias substitutas recebem essa denominação porque a artéria normal está ausente e porque podem suprir um lobo hepático inteiro sozinhas. Por outro lado, as artérias acessórias ou aberrantes são aquelas adicionais, não essenciais, funcionalmente responsáveis apenas por parte de um lobo^5^ .

No que concerne às suas implicações clínicas, complicações como necrose segmentar ou lobar hepática decorrentes da ligadura inadvertida de um desses vasos, principalmente das artérias substitutas, podem ser evitadas com o reconhecimento de tais variações anatômicas. Tal discernimento mostra-se ainda mais relevante nas videolaparoscopias, nos transplantes hepáticos de cadáveres ou intervivos e na incidência de gastroplastias nos últimos anos^3,5,6,22^ .

A incompreensão acerca das alterações do curso intra-hepático da VP pode prejudicar a técnica cirúrgica ou até mesmo inviabilizar cirurgias e procedimentos, como embolização de VP, hepatectomias, doação de órgão para transplante, entre outros^7,12,18,23^ .

Com relação ao uso dos exames de imagem para avaliação da anatomia hepática, os estudos morfológicos ou de imagiologia puderam aumentar a quantidade de participantes de pesquisas devido aos avanços tecnológicos dos métodos de imagem, uma vez que estes são mais rápidos que as técnicas de dissecação de cadáveres^6^ . Além disso, o uso dos exames possibilita um melhor planejamento de cirurgias hepáticas e, assim, a redução de taxas de morbimortalidade^4,22^ .

O padrão-ouro para avaliação de estruturas vasculares consiste na angiografia. Entretanto, o uso da tomografia computadorizada (TC) se mostra como uma alternativa veloz e de menor custo, obtendo, ainda, dados clínicos relevantes. Ademais, é possível aplicar diferentes técnicas, como o MIP (*maximum intensity projection*), o MPR (*multiplanar reconstruction*) e a reconstrução tridimensional, a fim de facilitar a caracterização dos vasos e, consequentemente, das variações anatômicas^9,12,19^ .

Devido à complexidade da irrigação hepática, a grande variabilidade arterial e venosa, a importância do acesso a esses vasos e a escassez de trabalhos correlacionando as estruturas avaliadas na TC trifásica, objetiva-se avaliar tanto a presença quanto a associação de ambas as variações hepáticas — arterial e portal — na população.

## METODOLOGIA

Trata-se de uma pesquisa retrospectiva de natureza básica e abordagem quantitativa descritiva, na qual foram avaliados 500 prontuários médicos de adultos brasileiros que realizaram exame de TC de abdome trifásico após adequada avaliação médica, por motivos não relacionados à pesquisa, em hospital da região meio-oeste do estado de Santa Catarina, Brasil, entre janeiro de 2021 e junho de 2022.

Este trabalho foi aprovado pelo comitê de ética em pesquisa sob o número de Parecer 5.616.116 e Certificado de Apresentação de Apreciação Ética 61836122.7.0000.5636, bem como concedida a dispensa do termo de consentimento livre e esclarecido. Como critérios de inclusão, foram considerados indivíduos com idade maior ou igual a 18 anos, de ambos os sexos, submetidos à TC abdominal por indicações clínicas variadas. Como critério de exclusão, foram considerados aqueles cujas anormalidades abdominais extremas dificultassem a avaliação da trama vascular do fígado.

Todas as imagens foram retiradas do arquivo pessoal dos autores, obtidas em equipamento de tomografia multidetectores de 16 canais, modelo Somaton Emotion (Siemens AG; Munique, Alemanha). O protocolo do exame consistia em cortes axiais de 1 mm, com *pitch* de 1,0. O contraste injetado foi o Omnipaque 350 (princípio ativo iohexol; GE Healthcare), na concentração de 755 mg/ml, injetado via intravenosa por meio de bomba injetora Medrad Vistron (Bayer), em fluxo de 3,5 ml/s, com tempo de retardo em *bolus tracking*. O *field-of-view* utilizado foi 250 mm, padrão. A espessura de reconstrução das imagens foi 1 mm. As imagens foram exportadas para o formato padrão DICOM e manipuladas utilizando os aplicativos de uso clínico Osirix MD (Pixeon, Genebra, Suíça) e RadiAnt (Medixant, Poznan, Polônia).

Analisou-se a anatomia de tronco celíaco, AGE, artéria esplênica, AHC, AHP, AHD, AHE, AMS, artérias hepáticas acessórias, VP, VP ramo esquerdo e VP ramo direito. As imagens foram classificadas por médico radiologista com 10 anos de experiência. Para o padrão arterial, foram realizadas análises no plano axial, técnicas de reconstruções no plano coronal e sagital em reconstruções MPR, assim como reconstruções tridimensionais em MIP.

Adotou-se como padrão de normalidade anatômica para o sistema arterial a AHP originando-se da AHC e bifurcando-se em hepáticas direita e esquerda, sem a presença de artérias acessórias para a vascularização arterial. À avaliação do sistema venoso, considerou-se a bifurcação do tronco principal da VP em ramos esquerdo e direito, com o último dividindo-se nos ramos direito anterior e direito posterior para a vascularização portal. As variações encontradas foram classificadas de acordo com Michels^14^ e Cheng et al.^24^ , respectivamente. Todos os dados observados foram manualmente organizados em tabelas no programa de planilhas Google Sheets, gerando gráficos automáticos pela plataforma que, consequentemente, foram analisados visualmente pelos pesquisadores.

## RESULTADOS

Em relação à caracterização da amostra, 307 indivíduos (61,4%) eram do sexo feminino e 193 (38,6%) do sexo masculino. A idade média dos participantes foi de 50,4±16,7 anos, com um mínimo de 18 e máximo de 92 anos. A anatomia referente à AH e suas variações foi classificada conforme estabelecido por Michels, em 1966 (Tabela 1), enquanto para as variações da VP hepática utilizou-se os critérios de Cheng et al.^24^ (Tabela 2).

**Tabela 1:** Anatomia da artéria hepática (AH) de acordo com Michels^14^ .

**Tipo**	**Descrição**	**Feminino**		**Masculino**		**Total**	
		**n**	**%**	**n**	**%**	**n**	**%**
I	Normal	207	67,6	137	71,4	344	68,8
II	AHE ramo AGE	17	5,6	7	3,6	24	4,8
III	AHD ramo AMS	18	5,9	9	4,7	27	5,4
IV	II e III	3	1,0	4	2,1	7	1,4
V	AHE acessória ramo AGE	22	7,2	19	9,9	41	8,2
VI	AHD acessória ramo AMS	7	2,3	4	2,1	11	2,2
VII	V e VI	0	0,0	1	0,5	1	0,2
VIII	III e V ou II e VI	11	3,6	7	3,6	18	3,6
IX	AHC ramo AGE	21	6,9	4	2,1	25	5,0
X	AHC ramo AMS	0	0,0	0	0,0	0	0,0

AHE = artéria hepática esquerda; AGE = artéria gástrica esquerda; AHD = artéria hepática direita; AMS = artéria mesentérica superior; AHC = artéria hepática comum. Fonte: Autores.

**Tabela 2:** Anatomia da veia porta de acordo com Cheng et al.^24^ .

**Tipo**	**Descrição**	**Feminino**		**Masculino**		**Total**	
		**n**	**%**	**n**	**%**	**n**	**%**
I	Bifurcação em ramos esquerdo e direito, com o último dividindo-se nos ramos anterior e posterior	237	77,5	153	79,7	390	78,2
II	Trifurcação da VP	17	5,6	13	6,8	30	6,0
III	Bifurcação precoce dos ramos anterior e posterior direitos, com VP esquerda originando-se do ramo posterior direito	25	8,2	13	6,8	38	7,8
IV	Ramo posterior direito originando-se do ramo esquerdo da VP	27	8,8	13	6,8	40	8,0

VP = veia porta. Fonte: Autores.

A distribuição anatômica normal da AH, ou Tipo I (Figura 1a) de Michels, que consiste na AHC emitindo a AHP, a qual bifurca-se em AHE e AHD, foi a preponderante neste estudo, correspondendo a 344 (68,8%) casos. O Tipo V (Figura 1e) foi o segundo mais predominante, sendo observado em 41 (8,2%) casos. Após, os Tipos III (Figura 1c), IX (Figura 1i) e II (Figura 1b) foram os mais presentes, com 27 (5,4%), 25 (5%) e 24 (4,8%) casos, respectivamente.

Nenhuma das amostras foi identificada com o Tipo X (Figura 1j), e dois (0,4%) participantes não puderam ser classificados. Um deles correspondendo a um caso de AHD com origem diretamente da aorta, e outro a um caso de AHC originando-se da AMS e AHE acessória originando-se da AGE, simultaneamente.

Sobre as variações venosas encontradas, 391 (78,2%) das tomografias apresentam VP intra-hepática de conformação normal conforme descrito no Tipo I de Cheng (Figura 2a). Os tipos IV (Figura 2d) e III (Figura 2c) obtiveram prevalência próxima, com 40 (8%) e 39 (7,8%) casos, respectivamente. Por fim, o Tipo II (Figura 2b) mostrou-se como o mais raro, com 30 (6%) ocorrências.

Quando analisamos a relação AH x VP, considerando toda a amostra, verificamos que a proporção de VP Tipo I (78,3%) (Tabela 2) é significativamente superior à AH Tipo I (69,1%) (Tabela 1), segundo o teste de qui-quadrado (p = 0,0009). Quando segmentamos a amostra por sexo, verificamos uma proporção não significativa para o sexo masculino (VP Tipo I, 79,7%, e AH Tipo I, 71,4%; p = 0,058) e significativa para o sexo feminino (VP Tipo I, 77,5%, e AH Tipo I, 67,6%; p = 0,006). Por fim, 258 (51,6%) participantes puderam ser classificados como anatomia normal tanto de AH quanto de VP, enquanto 33 (6,6%) obtiveram variação sincrônica de ambas.

## DISCUSSÃO

Demais estudos sobre a prevalência das variações da AH foram publicados (Tabela 3). O trabalho de Dazzi et al.^25^ avaliou uma amostra de 66 doadores de fígado para transplante, por meio de avaliação angiotomográfica e intraoperatória, revelando resultados com 100% de sensibilidade e 100% de especificidade. Os nossos resultados sobre a presença do Tipo I de Michels (68,8%) (Figura 3) são comparáveis com aqueles apresentados por Dazzi et al.^25^ , com 68,2%.

**Tabela 3:** Análise comparativa entre estudos acerca da prevalência das variações de artéria hepática.

**Classificação de Michels**		**Desta pesquisa (%)**	**Dazzi et al.** ^25^ **(%)**	**Fonseca-Neto et al.** ^8^ **(%)**	**Brasil et al.** ^4^ **(%)**	**Imam et al.** ^26^ **(%)**
I		68,8	68,2	86,8	82	60,9
II		4,8	10,6	2,7	1	9,1
III		5,4	10,6	5,6	10	7,4
IV		1,4	0	0,8	0	0,4
V		8,2	3	0,6	1	4,5
VI		2,2	0	0,4	1	0,4
VII		0,2	0	0,0	0	0,4
VIII		3,6	0	0,0	0	2,9
IX		5,0	3	0,0	4	0,8
X		0,0	0	0,0	0	0,0
Não classificável		0,4	4,5	2,9	1	12,9

Fonte: Autores.

**Figura 3:** Imagem de tomografia computadorizada de abdome com reconstrução em *maximum intensity projection* em fase arterial, plano coronal, demonstrando artéria hepática própria originando-se da artéria hepática comum, bifurcando-se em artérias hepáticas direita e esquerda.

Fonseca-Neto et al.^8^ , com base em 479 transplantes hepáticos, apresentou alta prevalência de anatomia normal (86,8%). Os Tipos II, III e IV foram encontrados em 2,7%, 5,6% e 0,8% dos casos, respectivamente, resultados semelhantes aos nossos, de 4,8%, 5,4% e 1,4%^8^ . Brasil et al.^4^ avaliaram 100 angiotomografias, encontrando prevalências de 1% para o Tipo VI e 4% para o Tipo IX. Esses números são similares aos 2,2% e 5% encontrados neste estudo.

Por fim, Imam et al.^26^ alcançaram resultados semelhantes àqueles que obtivemos quanto às alterações de AH referentes aos Tipos VII e VIII, com 0,4% e 2,9% respectivamente, enquanto no presente estudo as prevalências foram de 0,2% e 3,6%. Em contrapartida, observou-se uma alta incidência do Tipo V de Michels (Figura 4), com 4,5% da amostra, diferente da encontrada por nós, com 8,2%.

**Figura 4:** Imagem de tomografia computadorizada de abdome com reconstrução em *maximum intensity projection* em fase arterial, plano coronal, demonstrando artéria hepática esquerda acessória originando-se da artéria gástrica esquerda.

Das 500 TC analisadas nesta pesquisa, apenas duas (0,4%) não foram classificáveis conforme o modelo proposto por Michels. Outros estudos mostram uma falha na classificação de até 12,9%^26^ , conforme demonstrado na Tabela 3. Como as variações anatômicas derivam de alterações embriológicas, a existência de um modelo padronizado que aborde todas as possibilidades torna-se prejudicada^8^ .

Ambas as condições não classificáveis na presente pesquisa foram documentadas na literatura previamente, correspondendo aos casos de AHD originada diretamente da aorta e AHC originada da AMS com AHE acessória originada da AGE, simultaneamente. O primeiro caso mostra-se relevante devido ao difícil acesso, que demanda angiografia e quimioterapia transarterial para tratamento de hepatocarcinoma, enquanto o segundo está associado ao aumento das taxas de complicações no pós-operatório de pancreatoduodenectomia^26^ .

Realizando-se uma análise comparativa entre as pesquisas das variações de VP intra-hepática (Tabela 4), percebe-se que a nossa amostra obteve a maior incidência de variações da estrutura, com 21,8% dos exames de imagem analisados, contra 14,5% de Lee et al.^27^ , 4,8% de Anwar et al.^9^ , e 12,5% de Vidya et al.^28^ . Nossa amostra também apresentou a maior prevalência do Tipo IV de Cheng, com 8% dos casos, diferentemente dos últimos três estudos citados, com 1%, 0,8% e nenhum relato, respectivamente.

**Tabela 4:** Análise comparativa entre estudos acerca da prevalência das variações de veia porta intra-hepática.

**Classificação de Cheng**	**Desta pesquisa (%)**	**Lee et al.** ^27^ **(%)**	**Anwar et al.** ^9^ **(%)**	**Vidya et al.** ^28^ **(%)**
I	78,2	85,5	95,2	87,5
II	6,0	7,1	1,6	10,0
III	7,8	6,4	2,4	2,5
IV	8,0	1,0	0,8	0,0

Fonte: Autores.

A importância clínica referente à alta prevalência encontrada do Tipo IV de Cheng, de 1996 (Figura 5), baseia-se na sua contraindicação para certos procedimentos cirúrgicos, como doação de lobo hepático direito para transplante de órgãos ou ressecção de tumores, uma vez que impossibilita uma adequada reconstrução^9^ . Os estudos de Lee et al.^27^ e Vidya et al.^28^ obtiveram o Tipo II como o mais prevalente, com 7,1% e 10% dos casos, respectivamente. O estudo realizado por Anwar et al.^9^ apresentou como maior resultado os 2,4% de incidência do Tipo III.

**Figura 5:** Imagem de tomografia computadorizada de abdome em fase portal, plano coronal, demonstrando o ramo anterior direito da veia porta, originando-se do ramo esquerdo.

## CONSIDERAÇÕES FINAIS

Este estudo demonstra uma alta prevalência das variações na vascularização do fígado, tanto de AHP quanto de VP intra-hepática. Dados semelhantes foram encontrados em outros artigos globalmente. A análise tomográfica e o conhecimento sobre a anatomia vascular do fígado e de órgãos abdominais, tanto na sua forma clássica quanto variada, auxilia no melhor planejamento pré-operatório, maior sucesso cirúrgico, prevenção de complicações e redução da morbimortalidade. Variações não classificadas por ensaios prévios devem ser categorizadas conforme sua importância clínica, e mais estudos brasileiros devem ser encorajados, a fim de compreender melhor os padrões populacionais do país.
